# Comparison of effectiveness and safety of high-power vs. conventional-power radiofrequency ablation for treatment of atrial fibrillation

**DOI:** 10.3389/fcvm.2022.988602

**Published:** 2022-12-06

**Authors:** Penghui Cui, Yunpeng Qu, Jichang Zhang, Junduo Wu, Jing Zhang, Yongfeng Shi, Bin Liu

**Affiliations:** ^1^Department of Cardiology, The Second Hospital of Jilin University, Changchun, China; ^2^Department of Cardiology, Fuwai Hospital and Cardiovascular Institute, Chinese Academy of Medical Sciences and Peking Union Medical College, Beijing, China

**Keywords:** atrial fibrillation, catheter ablation, high-power, conventional-power, effectiveness and safety

## Abstract

**Aim:**

To compare high-power (HP) vs. conventional-power (CP) radiofrequency ablation for atrial fibrillation (AF).

**Methods:**

We retrospectively enrolled AF patients undergoing CP (30–40 W, 43 patients) or HP (50 W, 49 patients) radiofrequency ablation. Immediate pulmonary vein (PV) single-circle isolation, PV-ablation time, AF recurrence, AF recurrence-free survival, and complications were analyzed.

**Results:**

Diabetes was more common in the CP group than in the HP group (27.91% vs. 10.20%, *P* = 0.029). The left PV single-circle isolation rate (62.79% vs. 65.31%), right PV single-circle isolation rate (48.84% vs. 53.06%), and bilateral PV single-circle isolation rate (32.56% vs. 38.78%; all *P* > 0.05) did not differ between the groups. Single-circle ablation times for the left PVs (12.79 ± 3.39 vs. 22.94 ± 6.39 min), right PVs (12.18 ± 3.46 vs. 20.67 ± 5.44 min), and all PVs (25.85 ± 6.04 vs. 45.66 ± 11.11 min; all *P* < 0.001) were shorter in the HP group. Atrial fibrillation recurrence within 3 months (13.95% vs. 18.37%), at 3 months (11.63% vs. 8.16%), and at 6 months after ablation (18.60% vs. 12.24%; all *P* > 0.05) was similar in both groups. Atrial fibrillation recurrence-free survival did not differ between the groups (Kaplan-Meier analysis). Cardiac rupture and pericardial tamponade did not occur in any patient. Pops occurred in 2 and 0 patients in the HP and CP groups, respectively (4.08% vs. 0.00%, *P* = 0.533).

**Conclusion:**

High-power ablation improved operation time and efficiency without increasing complications.

## Introduction

Atrial fibrillation (AF) is a common tachyarrhythmia, with an increasing global prevalence. An estimated 43.6 million people worldwide suffered from AF/atrial flutter (AFL) in 2016 (1). The global prevalence rate of AF in adults is approximately 2–4% (1), with relatively higher prevalence rates in China, North America, Europe, and Southeast Asia (up to 270–360 cases per 100,000 people) (2). As population aging becomes increasingly prominent and medical tests continue to improve, it is estimated that by 2050, at least 72 million people in Asia and about 6 to 16 million people in the United States will be diagnosed with AF (3).

The harm of AF to humans should not be underestimated. Cardiogenic thromboembolism is one of the main causes of disability and death in patients with AF, and ischemic stroke is the most common consequence of cardiogenic thromboembolism, especially in patients with valvular AF (4). In addition, AF can increase the risk of heart failure and myocardial infarction, and patients with both heart failure and AF tend to have a higher risk of death (1). Thus, AF has become one of the greatest challenges of cardiovascular medicine in the twenty-first century. In 1997, Haissaguerre et al. (5) proposed that ectopic beats originating in the pulmonary veins (PVs) trigger AF. Based on this theory, it was proposed that radiofrequency ablation can be used to isolate the PVs and terminate AF, and indeed, promising clinical efficacy has been achieved with this method. In recent years, owing to in-depth research on the pathogenesis of AF, advances have been made in the technique of catheter ablation for the treatment of AF. The A4, SARA, CACAF, EAST-AFNET4, and other research trials have shown that catheter ablation is far superior to antiarrhythmic drugs for the treatment of AF (6–9). Therefore, the level of evidence for the recommendation of transcatheter ablation for AF in relevant guidelines and consensus statements has increased in recent years (1, 10–13). Currently, circumferential pulmonary vein isolation (CPVI) is the standard ablation procedure for AF.

Continuous, irreversible transmural damage is the key to successful ablation. Theoretically, under the conditions of stable abutment of the catheter with the myocardium and constant pressure, an increase in the output power and a decrease in the ablation time will increase the impedance heat and reduce the conduction heat, thereby increasing the continuity of the ablation focus and helping to improve PV-potential isolation (14). However, in the real world, it is unclear whether this high-power (HP) short-duration ablation strategy increases the incidence of operational complications such as pericardial tamponade and atrioesophageal fistula or improves the success rate and efficiency of the operation. Hence, the present real-world study aimed to comparatively analyze the efficacy and safety of HP vs. conventional-power (CP) radiofrequency ablation for the treatment of AF.

## Materials and methods

### Study population

This retrospective, single-center study included 100 patients with AF who underwent radiofrequency ablation in the Second Hospital of Jilin University between January 2019 and July 2021. The types of AF included paroxysmal atrial fibrillation (PAF; defined as AF duration ≤ 7 days) and persistent atrial fibrillation (PerAF; defined as AF duration >7 days). Of the 100 patients enrolled, 92 patients completed the follow-up assessments. The exclusion criteria were as follows: (1) patients aged ≥75 years; (2) patients with left atrial or left atrial appendage thrombosis confirmed by transesophageal echocardiography; (3) patients with acute pulmonary edema, acute hemorrhagic/ischemic stroke, or other diseases due to which they could not tolerate an operation; (4) patients with malignant tumor and a potential life span of < 1 year; (5) patients with severe bleeding tendency; (6) patients who were allergic to atropine, isoproterenol, or low-molecular-weight heparin; and (7) patients with incomplete operational data, laboratory data, or other clinical data and those who were lost to follow-up after the operation.

According to the power of radiofrequency ablation, patients were divided into a CP group (*n* = 43) and an HP group (*n* = 49). All patients signed informed consent forms before the operation. All patients underwent transesophageal echocardiography within 24 h before the operation to confirm that no thrombi existed in the left atrium or left atrial appendage. Patients with CHA_2_DS_2_-VASc scores ≥2 points were administered preoperative oral warfarin or a novel oral anticoagulant for ≥3 weeks. During oral warfarin treatment, the international normalized ratio was maintained at 2.0–3.0. Heparin bridging was not required before the operation, and oral anticoagulant drugs were discontinued in the morning on the day of the operation. Antiarrhythmic drugs such as dronedarone and propafenone were stopped at least five half-lives before the ablation. Fasting was required 6–8 h before the operation.

### Catheter ablation procedure

Before the operation, patients inhaled oxygen and were connected to an electrocardiogram (ECG) monitor. The operator disinfected the neck, chest, perineum, and inguinal area of the patient with iodophor. An in-dwelling urinary catheter was inserted. We used the CARTO 3 mapping system (BioSense Webster) for the operation. After successively puncturing the right femoral vein, left femoral vein, and right internal jugular vein under local anesthesia (1% lidocaine infiltration), the operator placed two 6F vascular sheaths, one 8F vascular sheath, and one 6F vascular sheath, respectively, in the above veins. Under X-ray guidance, a diagnostic 10-pole catheter was advanced into the coronary sinus through the right internal jugular vein, and a diagnostic four-pole catheter was placed in the right ventricle. Two transseptal punctures (SL1, 8.5F; Abbott) were performed under fluoroscopic guidance. The operator then injected heparin (80–100 IU/kg), and the activated clotting time was monitored and maintained between 300 and 350 s during the operation. The foramen ovale was then punctured in the same way as above, and an 8.5F puncture sheath was placed. A multielectrode mapping catheter (Pentaray NAV eco; BioSense Webster) and a contact force-sensing ablation catheter (ST/ST-SF; BioSense Webster) were delivered to the left atrium through an 8.5F SL1 sheath. The ablation procedures were guided by the CARTO 3 mapping system. The multielectrode mapping catheter was used to construct an anatomical model of the left atrium and the PVs. After the modeling was completed, the multielectrode mapping catheter was placed at the opening of the left inferior PV to adjust the respiratory gating. Pulmonary vein potentials were recorded throughout the operation. Under the power control mode, point-by-point circular ablation was performed using the contact force-sensing ablation catheter, starting from the rear walls of the left PVs and/or the anterior walls of the right PVs along the PV vestibule. The overlap between adjacent ablation points was about 10%, and the ablation procedure was usually enhanced by 3–4 points at the medial or lateral side of the PV crest on both sides. The end point of the ablation was the efferent block of the PV potentials as recorded by the multielectrode mapping catheter. Fentanyl (10–25 μg/kg) was given intravenously during the operation, and blood pressure, heart rate, and respiration were monitored throughout the operation. As the whole operation was performed under local anesthesia and with the patient in a conscious state, none of the patients underwent esophageal temperature monitoring during the operation.

In this study, all included patients were treated with radiofrequency catheter ablation under the guidance of an ablation index. In the CP group, ablation was performed using the ST ablation catheter. For ablation from the left atrium to the anterior walls of the PVs, the power was set to 35–40 W, with an average time of 20–30 s and a contact pressure of 10–15 g. The settings for ablation from the left atrium to the crests of the PVs were as follows: power, 30–40 W; average time, 30–35 s; and contact pressure, 10–15 g. For ablation from the left atrium to the posterior, inferior, and superior walls of the PVs, the following settings were used: power, 30–35 W; average time, 20–25 s; and contact pressure, 6–10 g (Table 1). In the HP group, ablation was performed using the ST-SF ablation catheter. The ablation power, time, and contact force settings for the left atrium to the anterior walls and crests of the PVs were 50 W, 15–20 s, and 7–15 g, and those for the left atrium to the posterior, inferior, and superior walls of the PVs were 50 W, 8–15 s, and 8–10 g (Table 1). All operations in both groups were performed in the power-control mode with an irrigation flow rate of 2 ml/min during mapping, 15 ml/min during ablation at 40–50 W, and 17 ml/min during ablation at 30–35 W. The Visitag (CARTO3; Biosense Webster) settings included: maximum displacement standard deviation, ≤ 2.5 mm; minimum time, 3 s; pressure, 5–20 g; maximal range, 2 mm; and interlesion distance, ≤ 6 mm.

**Table 1:** Ablation features.

	**Power/Time/Contact force/AI**	
	**CP group**	**HP group**
Anterior wall	35–40 W/20–30 s/10–15 g/500–550	50 W/15–20 s/7–15 g/500–550
Crest	30–40 W/30–35 s/10–15 g/500–550	50 W/15–20 s/7–15 g/500–550
Posterior wall	30–35 W/20–25 s/8–10 g/350–400	50 W/8–15 s/8–10 g/350–400
Inferior wall	30–35 W/20–25 s/6–10 g/400–450	50 W/8–15 s/8–10 g/400–450
Superior wall	30–35 W/20–25 s/6–10 g/450–500	50 W/8–15 s/8–10 g/450–500

CP, conventional power; HP, high power; AI, ablation index.

All patients underwent bilateral CPVI. Atrial flutter patients and PerAF patients received additional linear ablation, including mitral–isthmus linear ablation, tricuspid–isthmus linear ablation, left atrial roof linear ablation, left atrial–posterior box ablation, and matrix modification, and electrical cardioversion when necessary. For each PV, adenosine and isoproterenol were administered intravenously 30 min after successful isolation to confirm the bidirectional block of PV potentials. The above operations were performed by a single surgeon with more than 5 years of experience in electrophysiological operation in our center (Table 1).

### Post-ablation follow-up

All patients were instructed to fast for 2 h, stay in bed for 6 h, and have liquid food within 3 days after the operation. A normal 12-lead electrocardiogram (ECG) was reviewed immediately after the operation, and Holter ECG was reviewed on the second or third day after the operation. One of the following oral antiarrhythmic drugs was prescribed for 3 months after the operation: amiodarone 200–400 mg/d, propafenone 450 mg/d, or dronedarone 400 mg/bid, and the dose was adjusted according to the findings of outpatient reviews. Pantoprazole 40 mg/d or rabeprazole 10 mg/d was administered orally for 1 month after the operation, and hot or hard food was omitted for 30 days after the operation. Oral anticoagulation was continued at the patient's regular dose for at least 3 months depending on the CHA_2_DS_2_-VASc score.

The patients were followed up at 1, 3, 6, 12, and 18 months after the procedure. Electrocardiograms, Holter records, and echocardiography images were examined at all visits. Patients with any palpitation discomfort during this period could come to the hospital at any time. Clinical recurrence of AF was defined as the onset of atrial tachyarrhythmia, including AF, AFL, and atrial tachycardia (AT), with an ECG-recorded duration of ≥30 s occurring 3 months or more after the operation (12). Early recurrence of AF was defined as any ECG documentation of AT, AFL, or AF recurrence within 3 months after the procedure.

### Study outcomes

Procedural efficacy outcomes included PV single-circle immediate isolation rate, PV single-circle ablation time, lateral PV total ablation time, early AF recurrence rate, AF recurrence rate at 3 months after the operation, AF recurrence rate at 6 months after the operation, and long-term AF-free survival rate. It should be noted that bilateral PV single-circle isolation rate refers to the rate of successful immediate single-circle isolation of PV on both sides. Procedural safety outcomes included pops, pericardial effusion/tamponade, stroke/transient ischemic attack, thromboembolic events, PV stenosis, atrioesophageal fistula, phrenic nerve injury, and vascular complications such as arteriovenous fistula and pseudoaneurysm.

### Statistical analysis

Clinical data collection and postoperative follow-up were performed by the same author. Continuous variables with normal distribution were expressed as mean ± standard deviation, and compared using the Student *t*-test. Continuous variables with non-normal distribution were expressed as median and interquartile range, and compared using the Mann-Whitney *U*-test. Categorical variables were expressed as number and percentage, and compared using the χ^2^-test, Pearson chi-squared test, or Kruskal-Wallis test. The Kaplan-Meier survival estimate and log rank test of survival were used to evaluate the two ablation strategies at the endpoint of follow-up. All statistical analyses were performed using the SPSS statistical package *version* 22.0 (International Business Machines Corps). *P* < 0.05 was considered statistically significant.

## Results

### Baseline patient characteristics

A total of 92 patients were included in this study, with 43 patients in the CP group and 49 patients in the HP group. In the CP group, the mean age of the patients was 58.14 ± 9.83 years. The CP group included 34 (79.07%) male patients and 28 (65.12%) PAF patients. In the CP group, CPVI was performed using an ablation power of 35 W (anterior wall)/30 W (posterior wall) in 38 (88.37%) patients, 35 W (anterior wall)/35 W (posterior wall) in 2 (4.65%) patients, and 40 W (anterior wall)/35 W (posterior wall) in 3 (6.98%) patients. In the CP group, 3 (6.98%) patients had AFL, of whom, two underwent cavotricuspid–isthmus linear ablation and one underwent mitral–isthmus linear ablation. The 15 patients with PerAF also underwent electrical cardioversion.

In the HP group, the average age of the patients was 61.76 ± 9.45 years. The HP group included 39 male patients (79.59%) and 29 PAF patients (59.18%). All patients in the HP group underwent CPVI with an ablation power of 50 W (anterior wall)/50 W (posterior wall). Of the five patients (6.98%) with AFL, four underwent cavotricuspid–isthmus linear ablation, and one underwent mitral–isthmus linear ablation. One patient had AT originating from the right atrial septum; intraoperative mapping and ablation were performed for this patient. Two patients with PerAF recovered sinus rhythm during the operation, and the remaining 18 patients with PerAF underwent electrical cardioversion.

The general baseline data of the study subjects are shown in Table 2. The rate of diabetes was significantly higher in the CP group than in the HP group (27.91% vs. 10.20%, *P* = 0.029). There were no significant differences in age, sex, body mass index, AF type, other comorbidities, CHA_2_DS_2_-VASc score, HAS-BLED score, European Heart Rhythm Association (EHRA) class, postoperative medication, echocardiographic parameters, biochemical parameters, and ablation lesions between the two groups (Table 2).

**Table 2:** Baseline patient characteristics.

		**CP (*n* = 43)**	**HP (*n* = 49)**	***P***
Age (years)		58.14 ± 9.83	61.76 ± 9.45	0.076
Male sex, *n* (%)		34 (79.07)	39 (79.59)	0.530
BMI (kg/m^2^)		25.94 ± 3.34	25.55 ± 5.67	0.695
PAF, *n* (%)		28 (65.12)	29 (59.18)	0.559
Alcohol, *n* (%)		11 (25.58)	12 (24.49)	0.904
Smoking, *n* (%)		15 (34.88)	16 (32.65)	0.821
Comorbidities, *n* (%)				
	Diabetes	12 (27.91)	5 (10.20)	0.029
	Hypertension	24 (55.81)	18 (36.73)	0.067
	Hyperlipidemia	20 (46.51)	31 (63.27)	0.063
	Hyperuricemia	13 (30.23)	19 (38.78)	0.391
	CHD	10 (23.26)	10 (20.41)	0.741
	Heart failure	3 (6.98)	9 (18.37)	0.106
	Stroke	8 (18.60)	4 (8.16)	0.138
	Hypothyroidism	0 (0.00)	4 (8.16)	0.055
	AFL	3 (6.98)	5 (10.20)	0.859
	AT	0 (0.00)	1 (2.04)	1
CHA2DS2-VASc score		1 (1,2)	1 (1,2)	0.397
HAS-BLED score		1 (1,2)	1 (0, 2)	0.931
EHRA class, *n* (%)				0.376
	I	1 (2.32)	5 (10.20)	
	II	20 (46.51)	22 (44.90)	
	III	16 (37.21)	16 (32.65)	
	IV	6 (13.95)	6 (12.24)	
Postoperative medicine				
	β-Blocker	9 (20.93)	15 (30.61)	0.291
	ACEI/ARB	9 (20.93)	8 (16.32)	0.570
	ARNI	1 (2.32)	3 (6.12)	0.373
	Statins	16 (37.21)	13 (26.53)	0.137
Echocardiographic parameters				
	LAD, mm	38.40 ± 5.28	37.73 ± 0.87	0.583
	LVDs, mm	47.86 ± 5.61	48.73 ± 6.60	0.499
	LVEF (%)	62.40 ± 6.84	59.49 ± 10.23	0.109
Biochemical markers				
	eGFR (ml/min/1.73 m^2^)	86.55 ± 23.36	82.90 ± 19.93	0.420
	Cr (μmol/L)	79.87 ± 19.86	81.45 ± 22.41	0.726
	Uric acid (mmol/L)	381.58 ± 87.81	380.88 ± 121.08	0.975
	TC (mmol/L)	4.06 ± 0.88	3.96 ± 1.08	0.634
	LDL-C (mmol/L)	2.43 ± 0.71	2.57 ± 0.94	0.459
	TG (mmol/L)	1.61 ± 0.88	1.41 ± 0.78	0.239
Ablation lesion set, *n* (%)				
	CPVI ablation	43 (100)	49 (100)	1.00
	MI line	5 (11.63)	3 (6.12)	0.573
	CTI line	5 (11.63)	9 (18.37)	0.369
	LA top linear ablation	12 (27.91)	17 (34.69)	0.485
	LA matrix modification	2 (4.65)	6 (12.24)	0.358
	BOX ablation	4 (9.30)	9 (18.4)	0.213

CP, conventional power; HP, high power; BMI, body mass index; PAF, paroxysmal atrial fibrillation; CHD, coronary atherosclerotic heart disease; AFL, atrial flutter; AT, atrial tachycardia; ACEI, angiotensin-converting enzyme inhibitor; ARB, angiotensin receptor blocker; ARNI, angiotensin receptor-neprilysin inhibitor; EHRA, European Heart Rhythm Association; LA, left atrium; LAD, left atrial diameter; LVDs, left ventricular end-systolic dimension; LVEF, left ventricular ejection fraction; eGFR, estimated glomerular filtration rate; Cr, serum creatinine; TC, total cholesterol; LDL-C, low-density lipoprotein cholesterol; TG, triglycerides; CPVI, circumferential pulmonary vein isolation; MI, mitral-isthmus; CTI, cavotricuspid-isthmus.

### Procedural data

**Figure 1:**
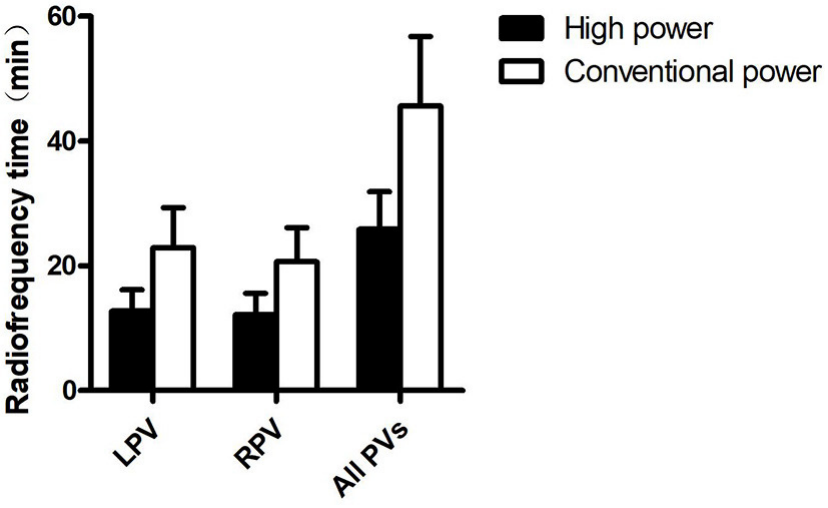
Ablation time. LPV, left pulmonary vein; RPV, right pulmonary vein; PV, pulmonary vein.

The results of PV single-circle isolation in the two groups are shown in Table 3. We found no statistically significant differences between the CP and HP groups in terms of the rates of single-circle isolation of the left PVs (62.79% vs. 65.31%, *P* = 0.802) or the right PVs (48.84% vs. 53.06%, *P* = 0.686). The bilateral PV isolation rate (32.56% vs. 38.78%, *P* = 0.535) also did not significantly differ between the two groups. The duration of ablation in the two groups is shown in Figure 1. Compared with the CP group, the HP group showed significantly shorter single-circle ablation times for the left PVs (12.79 ± 3.39 vs. 22.94 ± 6.39 min, *P* < 0.001), right PVs (12.18 ± 3.46 vs. 20.67 ± 5.44 min, *P* < 0.001), and all PVs (25.85 ± 6.04 vs. 45.66 ± 11.11 min, *P* < 0.001; Table 3, Figure 1).

**Table 3:** PV single-circle isolation rate.

	**CP (*n* = 43)**	**HP (*n* = 49)**	***P***
LPV single-circle isolation, *n* (%)	27 (62.79)	32 (65.31)	0.802
RPV single-circle isolation, *n* (%)	21 (48.84)	26 (53.06)	0.686
Bilateral PV single-circle isolation, *n* (%)	14 (32.56)	19 (38.78)	0.535

CP, conventional power; HP, high power; RPV, right pulmonary vein; LPV, left pulmonary vein; PV, pulmonary vein.

### Procedure-related complications

During the ablation procedure, pops were heard two times in total from two patients in the HP group, and were located at the top of the left superior PV and the top of the right superior PV. There were no serious consequences, such as cardiac rupture and pericardial tamponade, in these two patients. No pops occurred during the ablation procedure in the CP group. There was no significant difference in the incidence of pops between the two groups (4.08% vs. 0.00%, *P* = 0.533). No operational complications such as pericardial effusion/tamponade, stroke/transient ischemic attack, thromboembolic events, PV stenosis, atrioesophageal fistula, phrenic nerve injury, and vascular complications (e.g., arteriovenous fistula and pseudoaneurysm) occurred during the procedure.

### Follow-up outcomes

**Figure 2:**
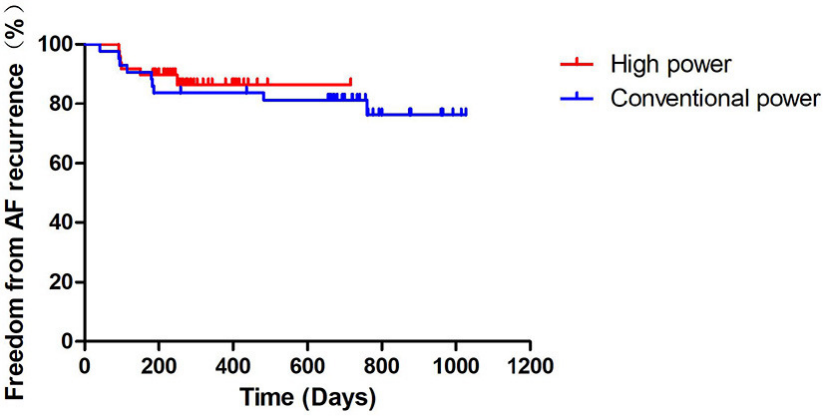
Kaplan-Meier curve of freedom from AF recurrence. AF, atrial fibrillation.

The recurrence rates of AF after the operation in the two groups are shown in Table 4. We found no significant differences between the CP and HP groups in terms of the early AF recurrence rate (13.95% vs. 18.37%, *P* = 0.567), and the clinical AF recurrence rates at 3 months (11.63% vs. 8.16%, *P* = 0.836) and at 6 months (18.60% vs. 12.24%, *P* = 0.397) after the operation. During a mean follow-up duration of 21.04 ± 9.01 months, there were a total of 15 recurrences (nine cases in the CP group and six cases in the HP). The Kaplan-Meier curves of AF-free survival did not significantly differ between the two groups (log-rank test: *P* = 0.622; Table 4, Figure 2).

**Table 4:** AF recurrence analysis.

	**CP (*n* = 43)**	**HP (*n* = 49)**	***P***
Early AF recurrence, *n* (%)	6 (13.95)	9 (18.37)	0.567
Postoperative AF recurrence at 3 months, *n* (%)	5 (11.63)	4 (8.16)	0.836
Postoperative AF recurrence at 6 months, *n* (%)	8 (18.60)	6 (12.24)	0.397

AF, atrial fibrillation; CP, conventional power; HP, high power.

## Discussion

### Main findings

The HP ablation strategy could effectively shorten the operation time and improve the operational efficiency of AF catheter ablation. The short-term and long-term efficacies of HP ablation were not inferior to those of CP ablation. Furthermore, HP ablation did not increase the incidence of complications of AF catheter ablation as compared with CP ablation, suggesting that the former procedure was safe.

### Immediate effectiveness of operation

Currently, radiofrequency ablation is an important method to maintain sinus rhythm in patients with AF, especially patients with symptomatic PAF. However, due to an incomplete understanding of the pathogenesis of AF and the limitations of the radiofrequency ablation technology, the current success rate of AF operations is far from perfect. In this regard, some authors have proposed that ablation be performed with increased radiofrequency output power and decreased point-by-point ablation times. This HP strategy may improve operational efficiency and success rate. An animal study has shown that ablation with output power/duration settings of 50 W/5 s and 60 W/5 s appeared to be more likely to achieve effective transmural lesions without increasing operational complications compared to the ablation settings of 40 W/30 s (15). Pambrun et al. (16) found that compared with low-power ablation (25–30 W), HP ablation (40–50 W) achieved a higher intraoperative initial PV single-circle isolation rate and a lower rate of intraoperative PV potential recovery, suggesting that the immediate effects of HP ablation may be better than those of low-power ablation. Okamatsu et al. (17) showed that compared with medium-power ablation (30–40 W) and low-power ablation (20–30 W), HP ablation (40–50 W) resulted in the highest rate of immediate single-circle isolation of the left and right PVs, with shorter ablation times for the left PVs, right PVs, and all PVs. Furthermore, the HP group had a lower rate of acute PV potential recovery. The study also confirmed that the HP ablation strategy may have higher immediate efficacy and efficiency than the low-power strategy. Berte et al. (18) showed that the total operation time and ablation time were significantly shorter in the HP group (35–45 W) than in the normal-power group (25–35 W), while the left and right PV single-circle isolation rates were similar. Yavin et al. (19) showed that compared with CP ablation (20–40 W/20–30 s), HP ablation (45–50 W/8–15 s) could significantly shorten the ablation time. Moreover, there were no statistically significant differences in the initial PV single-circle isolation rate between the above two groups, but the intraoperative acute PV potential recovery rate and the postoperative chronic PV potential recovery rate (defined as AF/AT occurrence for ≥30 s at 4 weeks after the operation) were significantly lower in the HP group than in the CP group. The results of the above study suggest that the HP ablation strategy can effectively shorten the operation time; its immediate operational effect is not inferior to that of the CP strategy, while its operation efficiency is higher.

In our study, the left, right, and bilateral PV single-circle isolation rates were similar in the CP and HP groups. However, compared with CP ablation, HP ablation significantly shortened the single-circle ablation times for the left PVs, right PVs, and all PVs. The HP ablation strategy mainly relies on the effect of impedance thermal damage. The ablation lesions are characterized by a shallow depth and wide range. In theory, this strategy can shorten the ablation time and improve the operational efficiency while ensuring effective transmural damage and continuity of the ablation point (20). The shortening of the ablation time can also reduce the amount of saline infusion to a certain extent, thereby reducing the amount of fluid intake, which is particularly important for patients with AF and heart failure. In conclusion, the results of this study suggest that the immediate operational efficacy of the HP ablation strategy is not inferior to that of the CP strategy, and that the HP strategy can significantly shorten the operation time and improve the operational efficiency.

### Short-term and long-term efficacy of operation

Some studies have shown that HP ablation (50 W) can achieve a right PV single-circle isolation rate of 88% and a left PV single-circle isolation rate of as high as 96%, with a relatively low intraoperative acute PV potential recovery rate (21). Kottmaier et al. (22) found that compared with CP ablation (30–40 W/20–40 s), HP ablation (70 W/5–7 s) could significantly reduce the AF recurrence rate at 1 year after the operation, suggesting that the long-term effect of HP ablation seems to be better than that of CP ablation. Baher et al. (23) reported that over a median follow-up duration of 2.5 years, similar AF-free survival rates were achieved using the HP strategy (50 W/5 s) and the low-power strategy ( ≤ 35 W/10–30 s). Yazaki et al. (24) reported that over a median follow-up duration of 2.5 years, similar rates of maintenance of sinus rhythm were achieved in the CP group (25–40 W) and the HP group (50 W), suggesting that the long-term outcomes of HP strategies are non-inferior to those of low-power strategies. Another study found that after HP ablation (50 W) for the treatment of AF, the sinus rhythm maintenance rate was approximately 86% at 1 and 2 years after the operation in PAF patients; for PerAF patients, these rates were 83% and 72%, respectively. Furthermore, Kaplan-Meier survival analysis showed that with a mean follow-up of 1.74 ± 0.61 years, the long-term AF recurrence-free survival rates of PAF and PerAF patients were similar, suggesting that the HP strategy is effective for both PAF and PerAF patients (25).

However, considering the transmurality of the lesions, the durability and stability of high-power short-duration (HPSD) ablation lesions is still under debate. Mohanty et al. (26) reported that among 1,749 patients with AF relapse after HPSD (45–50 W/5–15 s) ablation, electrical conduction reconnection/recovery mainly occurred in the coronal sinus (592, 40.8%), left atrial appendix (493, 34%), and PVs and left atrial posterior wall (249, 14.2%). In the above study, the ablation time during the prior HPSD ablation procedure was significantly shorter in the area facing the esophageal region than in other areas (5.2 ± 1.5 vs. 12.5 ± 1.7 s, *P* < 0.001), and the incidence of left atrial posterior wall reconnection in the area adjacent to the esophagus was 13.0% (227/1,749). In 73 patients, the esophagus was deflected using an esophageal displacement device during the prior HPSD ablation; among these patients, the average duration of ablation in the posterior wall was 9.2 ± 2 s, and PV-left atrial posterior wall reconnection was observed in 3 of the 73 patients (4.1%). The above study showed that at an output power of 45–50 W, an ablation time of 5 s is not enough to cause irreversible transmural injury to the posterior wall area facing the esophagus. Thus, the short ablation time of < 10 s was related to the partial myocardial conduction recovery in the coronary sinus, left atrial appendage, and left atrial posterior wall. Francke et al. (27) reported that among patients who underwent HPSD ablation (50 W; ablation index, 550 on the anterior wall and 400 on the posterior wall), the rate of first pass isolation of the left PVs was 95% and that of the right PVs was 92%. In addition, the acute PV potential reconnection rate of the bilateral PV carina was high, and it was necessary to supplement with point ablation again. During the blanking period, the AF/AFL/AT recurrence rate was 8%; during a follow-up period of 337 ± 134 days, the AF/AFL/AT recurrence rate was 3.75%. Hansom et al. (28) found that the incidence of no atrial arrhythmia was 79% in the HPSD group (anterior wall, 50 W/8–10 s; posterior wall, 50 W/6–8 s) and 73% in the low-power long-duration (LPLD) group, with no statistical difference (*P* = 0.339). During the follow-up period, 23 patients in the HPSD group and 29 patients in the LPLD group developed AF recurrence. Among patients who underwent a second operation, potential mapping showed that the right PV crest potential reconnection rate was higher in the HPSD group than in the LPLD group (46.7% vs. 20.6%; *P* = 0.035). This finding was related to lower catheter stability around right-sided veins. Other factors may further predispose the right PV carina for reconnection, such as increased tissue thickness and the presence of interatrial conduction tissue.

In our study, we found similar early AF recurrence rates, 3-month postoperative AF recurrence rates, and 6-month postoperative AF recurrence rates in the HP and CP groups. Kaplan-Meier survival analysis showed no statistically significant difference in the AF-free survival rate at the end of follow-up between the two groups. Continuous and uniform transmural injury is an important factor in determining the success rate of the operation, and it is affected by cardiac pulsation and respiratory movement. During the ablation process, the catheter will inevitably float and move in the cardiac cavity, making it difficult to stably stick to the myocardium. This unstable adhesion of the catheter with the myocardium is greatly increased during a long discharge, which makes it difficult to induce continuous and uniform transmural damage. The HP strategy can quickly generate ablation energy in a short period of time, shortening the ablation time and indirectly increasing the abutting stability between the catheter tip and the myocardium, which is helpful for the induction of continuous and uniform transmural damage and improves the success rate of the operation.

In conclusion, this study showed that the short-term and long-term efficacy of the HP ablation strategy was non-inferior to that of the CP ablation strategy, and that the HP ablation strategy was equally effective regardless of the type of AF.

### Operational safety

Pops, thromboembolism, cardiac effusion/tamponade, and atrioesophageal fistula are common complications of radiofrequency ablation for AF (29, 30). A study found that a total of four patients (8%) in the HP group (50 W/60 W) had pops, but none of them developed serious complications of cardiac rupture or pericardial tamponade; three patients (7%) in the low-power group (30 W) had pericardial tamponade events requiring urgent puncture treatment. Moreover, the frequency and severity of esophageal injury were significantly higher in the low-power group than in the HP group (*P* = 0.007), indicating that the safety of the HP short-duration ablation strategy seems to exceed that of the LPLD strategy (31). Another study reported one case (1%) of pericardial tamponade in the normal-power group (anterior, 35 W/posterior, 25 W) and no cases of pericardial tamponade in the HP group (anterior, 45 W/posterior, 35 W); this difference was not statistically significant. The overall incidence rate of operational complications was also similar between the normal-power and HP groups (1% vs. 3%, *P* = 0.39) (18). In one study, the incidence rates of pops during CPVI with the HP strategy (45–50 W/8–15 s) and CP strategy (20–40 W/20–30 s) were 0.07% and 0.03%, respectively, which were not significantly different. In addition, there were no operation-related complications such as pericardial tamponade and embolism in the two groups (19). The above results collectively show that the operational safety of the HP ablation strategy is not inferior to that of the CP strategy.

In our study, a total of two pops occurred during the operation in two patients in the HP group, one of which was located at the top of the left superior PV, and the other was located at the top of the right superior PV. The reason for the pops may be as follows: the tip of the ablation catheter was too tightly attached to the myocardial tissue during the operation, which instantaneously increased the pressure on the interface, and the saline perfusion was not smooth, which led to a sudden increase in the local temperature during discharge, resulting in a pop. Therefore, our center adjusted the pressure and maintained it in the range of 5–10 g, and pops did not occur again. In our study, there was no significant difference in the incidence of pops and other operational complications between the two groups (*P* > 0.05), indicating that the HP ablation strategy did not increase the incidence of operational complications.

## Conclusion

The HP ablation strategy for AF can effectively shorten the operation time and improve the operation efficiency without increasing the risk of operational complications.

## Limitations

The sample size of this study is relatively small. Due to incomplete operational data, this study did not further compare the immediate PV potential recovery rate during the operation. As endoscopy was not routinely performed after the operation, the differences in the severity of postoperative esophageal injury between the two groups also need to be further verified in order to more thoroughly assess the safety of the procedure.

## Data availability statement

The original contributions presented in the study are included in the article/Supplementary material, further inquiries can be directed to the corresponding author/s.

## Ethics statement

The studies involving human participants were reviewed and approved by the Second Hospital of Jilin University. The patients/participants provided written informed consent to participate in this study.

## Author contributions

All authors listed have made a substantial, direct, and intellectual contribution to the work and approved it for publication.

## Funding

This research was sponsored by the Education Department of Jilin Province (Grant No. JJKH20211197KJ) and Beijing United Heart Foundation (Grant No. 2021YX0117).

## Conflict of interest

The authors declare that the research was conducted in the absence of any commercial or financial relationships that could be construed as a potential conflict of interest.

## Publisher's note

All claims expressed in this article are solely those of the authors and do not necessarily represent those of their affiliated organizations, or those of the publisher, the editors and the reviewers. Any product that may be evaluated in this article, or claim that may be made by its manufacturer, is not guaranteed or endorsed by the publisher.
